# Supplementary material for: Identification of Freezing-Responsive microRNAs and Their Targets in Chinese Jujube by Small RNA and Degradome Sequencing
Source: Int J Mol Sci. 2026 Sep 4;27(17):7912. doi: 10.3390/ijms27177912 (PMC13566890; doi:10.3390/ijms27177912)
Supplement: Supplementary file 1 [file ijms-27-07912-s001.zip › Supplementary Material S2/novel_miR_130.pdf]

Provisional ID	:	novel_miR_130
Score total	:	0.3
Score for star read(s)	:	-1.3
Score for read counts	:	0
Score for mfe	:	0
Score for randfold	:	1.6
Score for cons. seed	:	
Total read count	:	93
Mature read count	:	93
Loop read count	:	0
Star read count	:	0

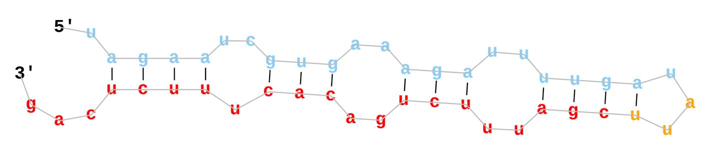

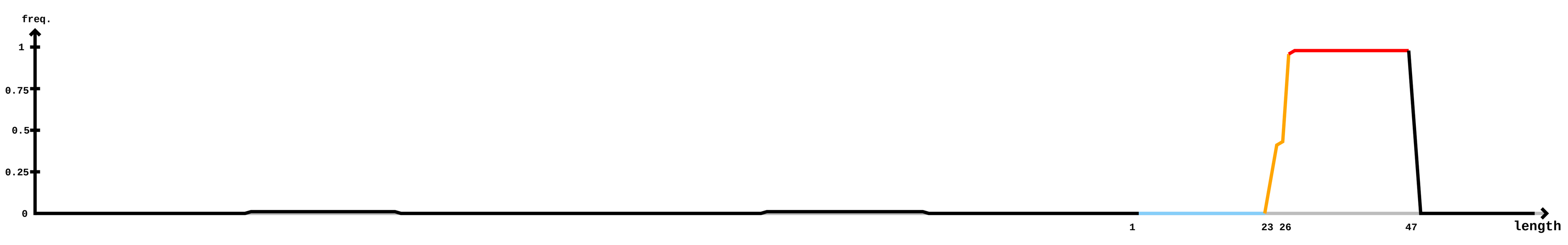

Star Mature

```
5'- uaaauuuucuaaauuaaauuucuauuguuuuguuuucuucuuuguuuggauuaguagcuguucaauuuaauuuaauuuaauuuauuauuauuauuaucauuguuauuguuuuuuagguuuugaugccuaagacaguaaauuuugaaauacacuguuuuuuugaguuuuuauuuuaucauuuuuagaaucgugaaagauuuugauauucgauuucugacacuuucucaggagccaaacggauagauaauuu -3'   exp
    .........(((((((((((...((((....(((....(((......))).....)))....))))..))))))))))).................. (((...((((((((..((((((......))))))))))))))....)))......((((((.((((.((((((.............((((..(((..(((..((((...))))..)))..))).)))).)))))))))).))))))........   reads   mm   sample
    ..................................uucuucuuuguuuggauuaguagcu................................................................................................................................................................................................   1   0   LT-1
    .........................................................................................................................ugaugccuaagacaguaaauuuugaaa.......................................................................................................   1   0   LT-2
    ..............................................................................................................................................................................................................uucgauuucugacacuuucucag......................   21   0   LCK-2
    ..............................................................................................................................................................................................................uucgauuucugacacuuucucag......................   18   0   LCK-1
    ...............................................................................................................................................................................................................ucgauuucugacacuuucucag......................   1   0   LT-1
    ...............................................................................................................................................................................................................ucgauuucugacacuuucucag......................   1   0   LT-3
    ................................................................................................................................................................................................................cgauuucugacacuuucucag......................   2   0   JCK-1
    ................................................................................................................................................................................................................cgauuucugacacuuucucag......................   17   0   LCK-2
    ................................................................................................................................................................................................................cgauuucugacacuuucucag......................   2   0   JCK-3
    ................................................................................................................................................................................................................cgauuucugacacuuucucag......................   1   0   LT-2
    ................................................................................................................................................................................................................cgauuucugacacuuucucag......................   1   0   JCK-2
    ................................................................................................................................................................................................................cgauuucugacacuuucucag......................   1   0   LT-1
    ................................................................................................................................................................................................................cgauuucugCcacuuucucag......................   1   1   JCK-2
    ................................................................................................................................................................................................................cgauuucugacacuuucucag......................   23   0   LCK-1
    ................................................................................................................................................................................................................cgauuucugacacuuucucagg.....................   2   0   LCK-3
    .................................................................................................................................................................................................................gauuucugacacuuucucag......................   2   0   LT-1
```
